# The Koreans

**Published:** 1904-07

**Authors:** 


### THE KOREANS.

The people of Korea are neither Japanese nor Chinese. They are Mongolians and have a polysyllabic language, with a phonetic alphabet. They have a recorded history of disputed authenticity which claims for them a continuous existence as a Korean people of about five thousand years, the earlier part of which of course is shrouded in mists of traditions and fable.

---

The *Boston Medical and Surgical Journal* publishes an important article by T. M. Rotch, M.D., in which the writer details all conditions to be met in gastro-enteritis and kindred diseases. This question is timely and of great importance as we near the season of dysentery and summer complaint. He shows that in "these diseases the gastric absorption is much interfered with, varying with the virulence of the disease and that the vomiting be associated with the acid intoxication found in these cases. In reducing this acidity he finds a remedy for the vomiting. Remedial measures at hand often fail in their purpose in consequence of their prompt ejectment by the stomach. This acid condition should be promptly met with some mild alkaline antiseptic. A combination of Glyco-Thymoline and Liq. Bismuth (equal parts) has been found most efficient. The intestinal tract in these cases is found intensely acid, characterized by foul irritating discharges tinged with blood and shreds of mucus, due to the process of putrefaction found especially in the colon. To check fermentation, and prevent the autoinfection, irrigation of the large intestine should be instituted. Glyco-Thymoline 3 i to the pint will be found to quickly reduce the temperature, tympanites and correct all fermentative processes. This treatment is nicely presented in a case recently occurring in the practice of J. F. Howe, M.D., of Brooklyn. Baby 15 months old, presented sudden fretfulness, drawing up of knees, tense knotted abdomen and considerable jactitation. Case resembled colic (enteritis). Vomiting and frequent diarrhea soon developed, stools a yellowish green, evidencing considerable intestinal irritation, great prostration, temperature 101° F. I ordered Glyco-Thymoline and Liq. Bismuth, equal parts to be given at first every two hours. Noted some blood streaks in passage and ordered a colon flush with a 10° solution of Glyco-Thymoline in warm water. After six hours there was evidence of a change. Child became more quiet, vomiting was checked. On the second day I found the passages reduced to one-half the number and on the fourth day the patient was practically well.

---

ONE of the most popular lawyers practicing at the local bar is James O'Shea, a Georgetown graduate, who has met with signal success at the trial table. Recently he represented a negro man charged in the police court with the larceny of several chickens. A fair defense was offered and things looked promising for the accused until the lawyer placed him on the stand. "Are you the defendant in this case?" said Judge Scott as the negro mounted the steps of the witness stand. "No, sir," replied the negro, with an amazed look on his face, and pointing to his counsel, "'Im the one that stole the chickens; there's the defendant."—*Washington Times.*

---

MRS. SMITH—"I'm afraid I shall have to stop giving Robby that tonic the doctor left him."

Mr. Smith—"Why, isn't he any better?"

Mrs. Smith—"Oh, yes. But he slid down the banisters six times this morning, broke the hall lamp, two vases, a jug, and a looking-glass, and I don't feel as if I could stand much more."—*Boston Traveler.*

Little Willie, who is a Philadelphia boy, had been watching a dog chasing his tail for three minutes.

"Papa," he asked, "what kind of a dog is that."

"That," said the father, "is a watch dog."

Willie was silent a moment. "Well," he finally said, "from the time he takes to wind himself up, I guess he must be a Waterbury watch dog."—*Detroit News-Tribune.*

---

"What are you going to do with all that patent medicine?" asked a man of a neighbor who was carrying half a dozen bottles.

"Wife's going to take it," he replied.

"Why," said the other in surprise, "I didn't know she was ill."

"Oh, she isn't," answered the party of the second part, "but she wants to get her picture in the papers. See?"—*Ex.*

---

**This Journal and Atlanta Semi-weekly Journal one year for $1.25 cash in advance. No checks. Strictly to new subscribers.**

---

Judge Henry Howland tells the story of the embarrassed but generous-hearted young man who felt called upon to relieve the sudden cessation of drawing-room conversation, which oftentimes overtakes even the most brilliant social circles. With the blushes surmounting his cheeks he timidly turned to the daughter of the hostess, who was not present in the room, and inquired:

"Ho-how is yo-your mo-mother? N-Not th-that I gi-give a——, but it ma-makes ta-talk."—*New Haven Register.*

---

A small boy in the primary grammar class, being told to compare the adjective "little," answered: "Little, small, nothing at all."—*Ex.*

